# Evaluation of anaesthesia and analgesia quality during disbudding of goat kids by certified Swiss farmers

**DOI:** 10.1186/s12917-018-1544-7

**Published:** 2018-07-09

**Authors:** N. Wagmann, C. Spadavecchia, U. Morath-Huss, G. Schüpbach-Regula, P. Zanolari

**Affiliations:** 1 Clinic for Ruminants, Vetsuisse Faculty, University of Bern, Bremgartenstrasse 109a, 3012 Bern, Switzerland; 2 Department of Veterinary Clinical Science, Anaesthesiology and Pain Therapy Division, Vetsuisse Faculty, University of Bern, Länggassstrasse 124, 3012 Bern, Switzerland; 3 Veterinary Public Health Institute, Vetsuisse Faculty, University of Bern, Schwarzenburgstrasse 155, 3097 Liebefeld, Bern, Switzerland

**Keywords:** Cautery disbudding, Goat kid, Analgesia, Anaesthesia, Switzerland

## Abstract

**Background:**

Certified Swiss farmers are allowed to disbud their goat kids using a standard intramuscularly administered anaesthetic mixture. This mixture, containing xylazine and ketamine, is officially distributed with the goal to provide a painless disbudding. This study aimed to evaluate the quality of analgesia and anaesthesia achieved during disbudding, when performed by Swiss farmers. To assess this, 174 goat kids at 31 different farms were observed and filmed during cautery disbudding.

**Results:**

The standard anaesthetic mixture (0.05 mg/kg xylazine and 20 mg/kg ketamine) was used only in 71 goat kids. Fifty-eight goat kids were anaesthetised with different dosages of xylazine (median 0.18 mg/kg) and ketamine (median 10 mg/kg), 22 with xylazine only (median 0.61 mg/kg), 20 with xylazine (median 1.84 mg/kg) and perineural lidocaine (median 1.23 mg/kg), three with acepromazine (dosage unknown) and ketamine (10 mg/kg). Based on vocalisation, limb movement and head lifting during disbudding, a general reaction score was attributed to 168 goat kids (six were excluded due to firm restraint): 56.5% were scored zero (no limb movement, no vocalisation), 7.7% one, 17.3% two and 18.5% three (strong movements, vocalisation). Significant risk factors for higher reaction scores were the type of anaesthetic protocol and manipulation by the farmer during induction. Significant risk factors for longer recoveries were use of xylazine alone or xylazine in combination with perineural lidocaine, breed, younger age and recovery underneath heat lamp.

**Conclusions:**

The present study indicates that anaesthesia and analgesia of goat kids disbudded by Swiss farmers is inadequate, as 35.8% of the animals showed moderate to strong behavioural reactions during the procedure. Unexpectedly, only 40.8% of the goat kids were anaesthetised with the standard anaesthetic mixture and several other protocols were used. A refinement of the recommended protocol is urgently needed to guarantee animal welfare.

**Electronic supplementary material:**

The online version of this article (10.1186/s12917-018-1544-7) contains supplementary material, which is available to authorized users.

## Background

In dairy goat farms, disbudding of goat kids is a common husbandry practice. While horn absence in adult animals is desirable to reduce fights and potential trauma to animals and humans, the removal of the horn buds is painful and stressful for the young goats [1, 2]. In Australia, New Zealand and the United States, disbudding of goat kids is routinely practised [3]. In Europe, procedures which cause a significant amount of pain or distress are in general forbidden, but exceptions for disbudding are made if allowed under existing national legislations. The European recommendation states that due to the anatomy of the kids’ skull, disbudding is a difficult procedure even if performed under anaesthesia; therefore, it should only be carried out by a veterinarian using an anaesthetic [4]. In Austria, disbudding is forbidden since 2005 [5], while in Germany and in the United Kingdom, disbudding can only be performed by a licensed veterinarian [6, 7].

In Switzerland, painful interventions can only be carried out under general or local anaesthesia by experienced personnel [8]. Swiss farmers who have acquired a certificate of competence by the Federal Food Safety and Veterinary Office (FSVO), are allowed to disbud their own goat kids under general anaesthesia until the age of 3 weeks. In order to obtain the certificate, the farmers are required to participate in a theoretical course, followed by practical experience under the supervision of a veterinarian at their own farm. The acquired skills are then evaluated by the cantonal veterinary inspection office and a certificate is issued [9].

Private veterinarians are expected to deliver a standard anaesthetic mixture to the certified farmers to anaesthetise their own goat kids for disbudding [10]. The standard mixture, foreseen by the FSVO for intramuscular administration, contains xylazine (0.05 mg/kg) and ketamine (20 mg/kg).

Since the introduction of the certificate course in 2008, no data have been collected to evaluate whether the procedure is carried out under adequate conditions for the animals. The main aim of the present study was to assess the quality of analgesia and anaesthesia of goat kids disbudded by certified Swiss farmers. Based on field observation, it was hypothesised, that anaesthesia quality might not be adequate for disbudding of young goat kids. To this end, several behavioural responses during disbudding were quantified through direct observation and later analysis of video recordings.

## Methods

### Study design and farm selection

The current study was designed as a prospective, observational field study. The Swiss Goat Breeding Association provided a list of 68 certified farmers that perform disbudding of goat kids themselves. These farmers were contacted by phone and were informed about the study. Thirty-one farmers were selected based on their agreement to participate in the study. Farmers performing disbudding with veterinary assistance were excluded from the study. Farm data and individual goat kid data were separately recorded. Farm data was collected prior to disbudding as well as goat kid data during disbudding.

### Farm data

Collected farm data included farm size, number of goat kids born per year, number of animals disbudded per year, average age at disbudding and past complications attributed to this intervention. Additionally noted was bedding type, heat lamp presence and infrastructure at the workplace.

Data collection at the farms took place from January to May 2017; time and date of disbudding were determined by the farmers. All farmers gave their informed written consent to have the disbudding procedure observed and recorded. Prior to disbudding, the farmers were interviewed using a standard questionnaire. Observation started at anaesthetic injection and ended when goat kids returned to a steady standing position after recovery from anaesthesia. Data were recorded with a standardized protocol (Additional files 1, 2). Farmers were asked to disbud their goat kids as per their routine farm procedure. There were two trained observers (NW and UM) and they did not interfere with the procedure at any stage. The video recordings^1^ were analysed at a later time point by NW and UM.

### Goat kid data

A total of 174 goat kids from 31 different farms were observed from the injection of the anaesthetic mixture to recovery. Individual goat kid data included ear tag number, breed, sex, age, weight, health condition and fasting duration. All goat-kids were observed from afar without being handled. Furthermore, composition, administration route and onset of action of the anaesthetic mixture, restraint method, skin preparation technique (clipping of the fur around the horn bud), disbudding technique (burning time, removing of horn bud, applying of dehorner), administration of analgesics and environmental temperature were recorded. To determine the dosage of drugs administered to goat kids of unknown body weight, a mean weight of 6.5 kg was assumed. During anaesthesia induction, time from injection to staggering, recumbency and loss of posture were measured. Recovery time was measured from the time from injection to first movement, to first attempt to stand and till steady standing (Additional file 3). Goat kids whose recovery could not be observed completely were excluded from further statistical analysis of recovery risk factors (eight for first movement, 22 for steady standing). The level of farmer care and monitoring of goat kids before and after disbudding were documented.

### Reactions to disbudding

Video recordings were analysed starting from the application of the dehorner and ended when cautery disbudding was finished. Specific behavioural reactions during disbudding were documented according to predefined events (Additional file 4). Struggles (movements of legs or attempts to escape), vocalisation and tail movements during cautery disbudding of goat kids are already described as signs of pain and stress [11, 12].

Frequency of occurrence of specific behaviours during disbudding was graded from 1 to 4 as follows: 1) behaviour did not occur; 2) behaviour occurred 1–2 times; 3) behaviour occurred 3–6 times; 4) behaviour occurred > 7 times.

Based on the occurrence of vocalisation, limb-movement (paddling/kicking/pull-up limb) and head lifting during disbudding, a general reaction score was attributed to each goat kid (Additional file 5) [13]. Firmly restrained animals were not scored, as behaviours could not be clearly observed.

Direct scoring of pain intensity during disbudding was performed using a Visual Analogue Scale (VAS) on a 10 cm line (0 meaning no pain, 10 meaning the worst possible pain) by the main investigator (NW) and, if wished, by the farmer.

### Risk factors

Most observed factors at farm and goat kid level (Additional file 6) were analysed to be potential risk factors for longer recovery and for higher general reaction score.

### Statistical analysis

A sample size of 166 animals was calculated to estimate the prevalence of reactions during disbudding with a precision of 7%, assuming a prevalence of 50%, a population size of 1082 disbuddings per year, and a 95% confidence.^2^ Data were recorded in Microsoft Excel spreadsheets.^3^ Descriptive analysis and screening of risk factors was performed with the statistics program NCSS10^4^ regression analysis with the software SAS 9.4.^5^ Variables that were recorded on a continuous scale were checked for normality with the Shapiro Wilk W test. Non-normal risk factor variables were grouped into biologically meaningful categories (Additional file 6). The effect of different anaesthesia mixtures (excluded the acepromazine group due to small number of animals) was analysed for three different outcomes (time to first movement, time to steady standing and general reaction score).Time to first movement and time to steady standing was recorded in minutes, and analysed as continuous, normally distributed variables. General reaction score was initially recorded on a 4-point scale and afterwards dichotomized into 0 (no reaction) and 1 (grade 1 to 3; Additional file 5).

For the screening of risk factors, ANOVA with Bonferroni correction for multiple comparisons was used for the outcomes time to first movement, time to steady standing and general reaction score. Associations with behavioural reaction during disbudding were screened with logistic regression. Potential risk factors which had a *P*-value < 0.1 in the screening were offered to a multiple linear or logistic regression model, respectively. None of the potential risk factors were correlated with each other (phi coefficients < 0.5). Generalized estimation equation was used to control for the effect of herd on the outcome [14]. SAS PROC GENMOD with herd as a REPEATED statement was used for this analysis. Variable selection was performed by stepwise backward selection until only significant (*P* < 0.05) risk factors remained in the model. No confounders (variables changing the effect of another risk factor by more than 20%) had to be included in the models. Two-way interaction terms had to be tested for significance but removed again from the final models to allow a more straightforward interpretation of the main effect of interest (anaesthesia protocol). Model fit was assessed using QIC and QICu. Model assumptions were checked by visual assessment of residuals versus predicted values. For the linear models, the assumption of normality of residuals was also formally checked with Shapiro Wilk W. Data were presented as median (range) or mean ± standard deviation (SD) unless otherwise specified.

## Results

### Farm data

#### Farm size

Herd size of included farms was 25 (4–260) goats that bred 35 (6–400) and disbudded 11 (2–80) goat kids per year. When asked about previously encountered adverse events during and after disbudding, 73.3% of the farmers reported to have never experienced any. The remaining reported cases from different farms of sudden death (6), excessive bleeding (2), prolonged recoveries (2), tetanus (1), split horn base (1) and intravenous injection of anaesthetics (1).

#### Infrastructure

For anaesthesia induction and recovery of goat kids 61.3% of the farmers utilized the same stalls; 38.7% had prepared separate places. A heat lamp was installed by 29.0% of the farmers at the induction area and by 41.9% at the recovery area. In 87.1% of the farms lighting was subjectively judged to be sufficient. Before disbudding, hair was clipped in 38.7% of the farms; 87.1% of the farmers used a disinfectant after disbudding, of which 74.1% contained an antibiotic substance (tetracycline or chloramphenicol). Environmental temperature during anaesthesia induction was 12.8 (4.0–25.0) °C and during recovery 14.7 (4.0–30.0) °C.

### Goat kid data

#### Animals

Goat breeds included Chamois-Coloured Goat (116), Saanen (39), Toggenburg (14) and Grisons Striped (5). Most animals were female (158), 12.0 (3.0–31.0) days of age with a body weight of 6.4 (3.0–11.0) kg. The kids (24.1%) were not weighed before disbudding; 95.4% of the goat kids had a healthy appearance, 3.4% showed signs of diarrhoea and 1.1% were lame. Fasting time was 4.0 (1.5–12.0) h; 19.5% of the goat kids were not fasted.

#### Administration of anaesthetic mixture

The recommended xylazine-ketamine mixture was used in 40.8% of the goat kids; different dosages of xylazine and ketamine were administered to 33.3%; 12.6% of the kids received only xylazine, 11.5% of the kids xylazine and local lidocaine; 1.7% of the kids were administered acepromazine combined with ketamine as anaesthetic protocol (Table 1). Only 6.3% of the goat kids from two different farms were administered an additional analgesic (meloxicam/ tolefenamic acid) after disbudding.

**Table 1:** Median (range) dosage of injected anaesthesia-mixtures by certified Swiss farmers to goat kids for horn disbudding

Number of goat kids	Xylazine dose (mg/kg)	Ketamine dose (mg/kg)	Lidocaine dose (mg/kg)	Acepromazine dose (mg/kg)	Mean general reaction score (0–3)
71	0.05^a^	20^a^	0		1.24 (SD 1.27)^b^
58	0.18 (0.05**–**0.58)	10 (4–25)	0		0.69 (SD 1.06)
22	0.61 (0.36**–**1.23)	0	0		1.55 (SD 1.34)
20	1.84 (0.61**–**1.84)	0	1.2 (1.2–3.1)		0.05 (SD 0.23)^c^
3	0	10	0	Dosage unknown	2.33 (SD 1.15)

^a^recommended xylazine-ketamine mixture

^b^mean of 66 goat kids (5 excluded due to strong restraint)

^c^mean of 19 goat kids (1 excluded due to strong restraint)

Injections were performed in the hindlimbs (M. semitendinosus/ M. semimembranosus or M. biceps femoris) in 59.2% of the goat kids and in the forelimbs (M. supra−/infraspinatus, *M. deltoideus*, M. brachiocehalicus or M. omotransversarius) in 40.8%. Aspiration before injection was performed in 29.3% of the animals. No reaction at injection was observed in 69.5% of the kids. Only few animals showed vocalisation (12.1%) and limb movement (5.7%) or both at the same time (12.6%). Waiting time from injection to disbudding was 12 (3–46) min. Two of the 31 farmers used lidocaine for local anaesthesia. They inserted the needle close to the caudal ridge of the frontal process of the zygomatic bone to block the cornual branch of the zygomaticotemporal (lacrimal) nerve.

#### Anaesthesia induction phase

Farmers (90.3%) observed the goat kids until loss of posture, while 6.5% observed the animals at 5 min intervals. One farmer left the animals alone after the anaesthetic injection until disbudding was started. During anaesthetic induction, 40.8% of the goat kids were manipulated by the farmers before loss of posture. Induction times were 1.5 (0–5) min for staggering; 2.0 (0–13) min for sternal recumbency, 3.0 (1–13) min for lateral recumbency and 3.0 (1–13) min for loss of posture.

#### Recovery phase

Only 25.8% of the farmers observed their goat kids during the entire recovery phase; 35.8% sporadically looked after them (at minimum every 15 min), and 38.7% of the farmers did not observe the goat kids during recovery at all.

During the recovery phase, 38.5% of the goat kids were touched by the farmers, while 61.5% were left completely undisturbed. Overall, time from injection to first movement was 49.4 ± 27.6 min and to steady standing was 108.7 ± 45.2 min. Time to first movement for goat kids anaesthetised with the official ketamine-xylazine dosages was 35.3 ± 18.9 min and to steady standing 82.3 ± 26.0 min.

### Reactions to disbudding

#### Video analysis

Cautery disbudding techniques varied: in 71.3% of the goat kids, a hot dehorner was rotated to burn larger circles around the horn bud region and in 28.7% of the animals it was held steadily, perpendicular to the skull. In 18.4% of the goat kids a pen knife or shears were used to remove the tip of the horn bud before cautery, depending on the size of the horn buds. After burning, the isolated central circle containing the horn bud was removed in 37.9% of the goat kids, while the remaining farmers left the bud in place. Burning time ranged from 7 to 120 s per horn (median 25 s), with the hot iron being applied between 1 to 35 times per horn (median 3 times). 25.8% of the farmers left the dehorner longer than 60 s per horn on the kid’s heads. The use of restraints for disbudding, rendering the animal immobile was applied in 20.1% of the animals, other farmers just held the head of the goat kids. Due to strong reactions, three farmers decided to administer an additional dosage of anaesthetic to five goat kids during disbudding, three of them were anaesthetised with the recommended mixture and two with xylazine alone (additional dosage: once 0.3 mg/kg xylazine, once 0.17 mg/kg xylazine and three times a mixture of 0.01 mg/kg xylazine plus 3.5 mg/kg, 3.7 mg/kg, 4.3 mg/kg ketamine).

Frequency of occurrence of specific behaviours is presented in Table 2. General reaction scores during disbudding could be evaluated for 168 goat kids: score 0 was attributed to 56.5% of the animals, score 1 to 7.7%, score 2 to 17.3% and score 3 to 18.5%.

**Table 2:** Number (n) of goat kids showing the following reactions during the disbudding procedure (Na = not adjustable)

Event	Grade 1 (0)	Grade 2 (1–2)	Grade 3 (3–6)	Grade 4 (> 6)	Na
Head lifting (n)	153 (88.4%)	5 (2.9%)	4 (2.3%)	6 (3.5%)	5 (2.9%)
Paddling (one limb) (n)	159 (91.9%)	8 (4.6%)	1 (0.6%)	0	5 (2.9%)
Paddling (several limbs) (n)	152 (87.9%)	5 (2.9%)	8 (4.6%)	3 (1.7%)	5 (2.9%)
Kicking (one limb) (n)	161 (93.1%)	5 (2.9%)	1 (0.6%)	1 (0.6%)	5 (2.9%)
Kicking (several limbs) (n)	162 (93.6%)	4 (2.3%)	2(1.2%)	0	5 (2.9%)
Pull up (one limb) (n)	136 (78.6%)	23 (13.3%)	9 (5.2%)	0	5 (2.9%)
Pull up (several limbs) (n)	154 (89.0%)	13 (7.5%)	1 (0.6%)	0	5 (2.9%)
Focused eye movement (n)	114 (65.9%)	11 (6.4%)	6 (3.5%)	1 (0.6%)	41 (23.7%)
Spontaneous blinking (n)	49 (28.3%)	32 (18.5%)	24 (13.9%)	27 (15.6%)	41 (23.7%)
Ear movement (n)	131 (75.7%)	8 (4.6%)	1 (0.6%)	0	33 (19.1%)
Tail movement (n)	87 (50.3%)	34 (19.7%)	14 (8.1%)	10 (5.8%)	28 (16.2%)
Nose Movement (n)	132 (76.3%)	3 (1.7%)	0	0	38 (22.0%)
Mouth Movement (n)	124 (71.7%)	8 (4.6%)	3 (1.7%)	1 (0.6%)	37 (21.4%)
Groaning (stimulus-associated) (n)	96 (55.5%)	28 (16.2%)	24 (13.9%)	25 (14.5%)	0
Vocalisation (n)	108 (62.4%)	17 (9.8%)	21 (12.1%)	27 (15.6%)	0

#### VAS-scoring

Mean VAS attributed by the observer was 3.2 ± 3.1. Farmer VAS was 2.1 ± 2.4. Twenty-seven farmers did not score a VAS. For both scores, values ranged between 0 and 10.

### Risk factors

#### Longer recovery

Goat kids anaesthetised with non-standard protocols required longer time from injection to first movement and longer to stand. Furthermore, breed and age had a significant effect on recovery as younger goat kids (≤14 days) needed longer to stand than older ones. Goat kids recovering under a heat lamp needed longer to stand steady than goat kids recovering without heat lamp (Tables 3, 4). Goat kids (5.7%) showed head rubbing on objects or companions during the recovery phase.

**Table 3:** Analysis of risk factors for first movement after disbudding in goat kids anaesthetised with different protocols by certified Swiss farmers. Final generalized estimation equation model correcting for the effect of the herd, *n* = 30 herds and 166 goat kids (CI, confidence interval; Ref, reference). Eight goat kids were excluded, because first movement could not be exactly observed

Risk factor	Beta (in minutes)	95%CI	*p*-Value
Anaesthesia protocol			< 0.001
Recommended xylazine-ketamine mixture	Ref	Ref	Ref
Xylazine-ketamine (other dosage)	7.0	−4.2-18.1	0.223
Xylazine	17.4	1.4–33.4	0.033
Xylazine-Lidocaine	50.2	40.4–59.9	< 0.001
Breed			< 0.001
Chamois-Coloured	Ref	Ref	Ref
Saanen	20.7	8.3–33.1	0.001
Toggenburg	18.6	7.9–29.3	< 0.001
Grisons Striped	−7.1	−25.7-11.5	0.454

**Table 4:** Analysis of risk factors for being able to stand steady after disbudding in goat kids anaesthetised with different protocols by certified Swiss farmers. Final generalized estimation equation model correcting for the effect of the herd, *n* = 29 herds and 152 goat kids (CI, confidence interval; Ref, reference). Twenty-two goat kids were excluded, because recovery could not be visited to the end

Risk factor	Beta (in minutes)	95%CI	*p*-Value
Anaesthesia protocol			< 0.001
Recommended xylazine-ketamine mixture	Ref	Ref	Ref
Xylazine-ketamine (other dosage)	14.7	−6.0-35.3	0.165
Xylazine	71.7	49.5–93.8	< 0.001
Xylazine-Lidocaine	62.1	41.4–82.8	< 0.001
Breed			0.008
Chamois-Coloured	Ref	Ref	Ref
Saanen	−20.2	−41.0-0.7	0.058
Toggenburg	13.3	−8.0-34.6	0.222
Grisons Striped	24.9	1.4–48.4	0.038
Age of goat kids			< 0.001
> 14 days	Ref	Ref	Ref
≤ 14 days	17.7	8.9–26.4	< 0.001
Heat lamp in recovery box			0.008
No	Ref	Ref	Ref
Yes	28.4	7.6–49.3	0.008

#### Higher reaction grade

The anaesthetic mixture and manipulation of all animals during anaesthesia induction were identified as significant risk factors for behavioural reactions during disbudding (limb movement, head lifting, vocalisation). Goat kids anaesthetised with xylazine followed by lidocaine infiltration had a 10 times lower risk to show reactions than goat kids anesthetised with the recommended xylazine-ketamine mixture. Goat kids being manipulated during anaesthesia induction (before loss of posture) showed 3.5 times more reactions than goat kids left undisturbed (Table 5).

**Table 5:** Analysis of risk factors for limb movement, head lifting and/or vocalisation in goat kids anaesthetised with different anaesthesia protocols for horn disbudding. Final generalized estimation equation model correcting for the effect of the herd, n = 30 herds and 165 goat kids (CI, confidence interval; Ref, reference). The three goat kids of the acepromazine-ketamine group were excluded (to small group for statistical analysis). Six goat kids of the other four groups were excluded due to firm restraint

Risk factor	Odds Ratio	95%CI OR	*p*-Value
Anaesthesia protocol			0.003
Recommended xylazine-ketamine mixture	Ref	Ref	Ref
Xylazine-ketamine (other dosage)	1.1	0.3–3.4	0.915
Xylazine	1.8	0.3–9.4	0.503
Xylazine-Lidocaine	0.1	0.0–0.3	< 0.001
Intervention of goat owner during induction			0.002
No	Ref	Ref	Ref
Yes	3.5	1.6–7.9	< 0.001

## Discussion

Aim of this study was to assess the quality of analgesia and anaesthesia of goat kids disbudded by certified Swiss farmers. The findings show clearly that there is a need to improve the anaesthetic protocol used by certified Swiss farmers to anaesthetise their goat kids for disbudding. Nearly half of the goat kids (43.5%) had a general reaction score of 1–3 and therefore showed signs of consciousness or pain during the disbudding procedure; 18.5% of the goat kids had a general reaction score of 3, indicating strong movements and vocalisation.

The anaesthetic protocols used for disbudding varied significantly and were often not consistent with the official recommendations. In 2008, when the farmers certificate of competence for disbudding of own goat kids was introduced, the FSVO recommended to use a mixture of xylazine (0.05 mg/kg) and ketamine (20 mg/kg) based on an unpublished field study on 40 goat kids. At that time, this combination seemed to represent the best compromise for a good quality anaesthesia and reasonable recovery times, using drugs approved for use in goats. In the literature, only little information is available about anaesthesia of goats in general and in particular of goat kids. Most of the recommendations are inferred from experience in sheep. A mixture of xylazine (0.1–0.2 mg/kg) and ketamine (10 mg/kg) to be administered intramuscularly is reported for the use in goats [15]. While the ketamine doses used by the farmers, in the present study, were in the range of the above reported doses, xylazine was overdosed in several occasions. According to the farmers, four veterinarians seemed to refuse to provide ketamine to the farmers. This probably led to the use of increased xylazine doses (up to 1.84 mg/kg). This dose represents almost 20 times the recommended dose for goats (0.1 mg/kg IM or 0.05 mg/kg IV), that are considered more sensitive to xylazine than sheep [16]. Accidental intravenous injection could not be ruled out if aspiration was not performed (70.3%).

A study on adult goats reported undesired cardiopulmonary effects such as hypoxemia, hypotension, hypoventilation and bradycardia following administration of intravenous xylazine (0.05 mg/kg) [17]. Clearly, side effects such as deep anaesthesia and long duration of action have to be expected after administration of the high doses used in the present study. Indeed, in those animals, complete absence of reaction to disbudding indicating deep anaesthesia and prolonged recoveries were observed. Surprisingly, goat kids recovering underneath a heat lamp took a longer time to steady standing. The improved cardiovascular and metabolic state should lead to a faster recovery from anaesthesia, but as the animals were left completely undisturbed, they might have felt comfortable underneath the heat lamp and they remained lying.

Goat kids that were administered xylazine followed by a lidocaine nerve block showed less behavioural reactions than goat kids anaesthetised with the other four anaesthetic protocols. Dosages of 1.2 mg/kg (0.2 ml lidocaine 2% per horn) and 3.1 mg/kg lidocaine (0.5 ml lidocaine 2% per horn) were used, respectively. Both farmers using lidocaine for local anaesthesia blocked only the cornual branch of the zygomaticotemporal (lacrimal) nerve. In goats, the sensory innervation of the horn is also provided by the cornual branch of infratrochlear nerve that can be blocked at the dorsomedial margin of the orbit [15]. Alvarez et al., 2015 showed that the cornual nerve block with lidocaine did not prevent stress (measured with cortisol-levels) and painful reactions such as vocalisation in goat kids during and after disbudding, even in higher dosages of 1 ml 2% lidocaine per horn [18]. Therefore, the most probable reason for the reduced reactions of goat kids disbudded under systemic xylazine and local lidocaine in this study was the high xylazine dose and not the local anaesthesia.

Further discussion point is the insufficient analgesia after the disbudding procedure. Non-steroidal anti-inflammatory drugs (meloxicam or tolefenamic acid) were administered to only 11 goat kids. Administration of meloxicam significantly reduced signs of pain, as measured with a VAS, in goat kids on the first day after disbudding [19]. One reason for the scarce administration of analgesics are the higher costs.

Not only the anaesthetic protocol, but also the disbudding technique varied considerably. Burning time per horn differed strongly (7–120 s). Short time application of the dehorner (< 20–25 s in total) and a proper disbudding technique are necessary, because the skull of goat kids is much thinner and the horn bud larger than in calves [20, 21]. The thin skullcap and the small sinus frontalis facilitate complications during thermal disbudding, such as necrosis of the frontal bone and the underlying frontal cortex of the brain [22]. A standard and well-defined disbudding technique will be necessary for future studies on anaesthesia and analgesia refinement. Further important point is a solid education of farmers to ensure adequate disbudding technique and proper administration of anaesthetics, what has to be realised by the FSVO.

Limitations of the present study were the small number of farmers actually disbudding without assistance of a veterinarian and therefore the limited number of observable animals. The influence of the various anaesthetic protocols was large due to the small sample size, so that many other possible risk factors for higher general reaction score (as for example waiting time from injection to disbudding, burning time or experience of disbudder by number of disbudded goat kids per year) did not become significant. Further limitations were the voluntary participation in the study and the observation at non-standardized field condition.

## Conclusions

Anaesthesia and analgesia quality during disbudding performed by certified Swiss farmers was not sufficient, even in cases in which the officially recommended ketamine-xylazine mixture was administered. From the point of view of animal welfare, further studies to develop appropriate and safe anaesthesia protocols for young goat kids are definitely required, as well as improvement of farmer education to ensure proper administration of anaesthetic mixtures.

## Additional files

Additional file 1: Data collection recorded with a standardized protocol (goat kid protocol). Includes the goat-kid protocol (DOCX 43 kb).

Additional file 2: Data collection recorded with a standardized protocol (farm protocol). Includes the farm protocol (DOCX 38 kb).

Additional file 3: Description of the behavioural states and events observed during anaesthesia induction and recovery of goat kids before and after disbudding. Describes the following behaviours: Staggering, recumbency, loss of posture, first movement, attempt to stand, steady standing (DOCX 36 kb).

Additional file 4: Description of behavioural events observed during disbudding of anaesthetised goat kids. Describes the following events: Head lifting, limb movement, focused eye movement, spontaneous blinking, ear movement, tail movement, nose movement, mouth movement, vocalisation (DOCX 37 kb).

Additional file 5: General reaction score of goat kids during disbudding based on vocalisation, limb movement and head lifting. A score between 0 and 4, zero means no movement and no vocalisation, four means strong movements and vocalisation (DOCX 36 kb).

Additional file 6: Risk factors for the three outcomes grade, first movement and steady standing, divided in farm-level risk factors and goat kid-level risk factors. Description and categorization of the different risk factors on farm and goat kid level (DOCX 38 kb).

## Abbreviations

ANOVA: Analysis of variance
FSVO: Federal Food Safety and Veterinary Office
NW, UM: N. Wagmann, U. Morath-Huss
QIC/ QICu: Quasi-likelihood under the independence model criterion
SD: Standard deviation
VAS: Visual Analogue Scale

## Footnotes

1 GoPro Hero4 silver edition camera (https://gopro.com/update/hero4)

2 WinEpiscope 2.0, Software for Quantitative Veterinary Epidemiology, Facultad de Veterinaria, Zaragoza, Spain, Agricultural University Wageningen, the Netherlands, University of Edinburgh, UK (http://www.winepi.net)

3 Microsoft® Office Excel 2016, Microsoft Corporation (https://www.microsoft.com)

4 NCSS 10 Data Analysis, Statistical Software, Kaysville, Utah, USA (https://www.ncss.com)

5 SAS® 9.4 Software (http://support.sas.com/software/94/index.html)
