# Supplementary material for: Evaluation of anaesthesia and analgesia quality during disbudding of goat kids by certified Swiss farmers
Source: BMC Vet Res. 2018 Jul 9;14:220. doi: 10.1186/s12917-018-1544-7 (PMC6038348; doi:10.1186/s12917-018-1544-7)
Supplement: Supplementary file 1 — Data collection recorded with a standardized protocol (goat kid protocol). Includes the goat-kid protocol (DOCX 43 kb). [file 12917_2018_1544_MOESM1_ESM.docx]

***Additional file 1***

Data collection recorded with a standardized protocol (goat kid protocol).

Goat kid protocol

| ear tag number |  |
| --- | --- |
| breed |  |
| age (days) |  |
| weight |  |
| health condition |  |
| fasting duration |  |
| gender |  |

Preparations

| dehorner (DM, warmed up?) |  |
| --- | --- |
| anaesthesia und analgesia drugs (which ones) |  |
| disinfectant |  |
| clipping |  |

**Administration of the anaesthetic mixture**

| location (environment) |  |
| --- | --- |
| anaesthetics   - (1) dose (ml/kg or mg/kg) - (2) concentration (mg/ml) |  |
| restraint method |  |
| handling with the animals (calm?) |  |
| injection   - (1) time - (2) localisation - (3) aspiration (yes/no?) - (4) reaction (yes/ no, what?) |  |
| body temperature of goat kid (time) |  |
| environmental temperature |  |

**Anaesthesia induction phase**

| surveillance? |  |
| --- | --- |
| intervention of the farmer? manipulation? |  |
| staggering (time) |  |
| recumbency (time)   - (1) sternal - (2) lateral |  |
| loss of posture (time) |  |

**Transport**

| time |  |
| --- | --- |
| reaction during transport (yes/ no, what?) |  |
| placement on table (time) -> reaction? |  |
| - head lifting |  |
| - vocalization |  |
| - limb movement   - paddling   - kicking   - pull up limb |  |
| - focused eye movement |  |
| - spontaneous blinking |  |
| - ear movement |  |
| - tail movement |  |
| - nose movement |  |
| - mouth movement   (teeth grinding, chewing) |  |

**The disbudding procedure**

| restraint of the goat kid |  |
| --- | --- |
| lateral position (right/left/sternal) |  |
| clipping around horn bud   - time/ reaction? what? |  |
| technique   - burning time - removing of the horn bud - applying of the dehorner (rotation? How many times per horn) |  |
| disinfection (time) |  |
| additional dosage of anaesthetics   - (1) time - (2) dose - (3) localisation - (4) waiting time |  |
| environmental temperature |  |

1. Horn

| side (left or right?) |  |
| --- | --- |
| start time |  |
| - head lifting |  |
| - vocalization |  |
| - limb movement   - paddling   - kicking   - pull up limb |  |
| - focused eye movement |  |
| - spontaneous blinking |  |
| - ear movement |  |
| - tail movement |  |
| - nose movement |  |
| - mouth movement   (teeth grinding, chewing) |  |
| end time |  |

2. Horn

| side (left or right?) |  |
| --- | --- |
| start time |  |
| - head lifting |  |
| - vocalization |  |
| - limb movement   - paddling   - kicking   - pull up limb |  |
| - focused eye movement |  |
| - spontaneous blinking |  |
| - ear movement |  |
| - tail movement |  |
| - nose movement |  |
| - mouth movement   (teeth grinding, chewing) |  |
| end time |  |

**After disbudding**

| transport to recovery area (time/ reactions?) |  |
| --- | --- |
| stimulation/ manipulation by farmers (time)? |  |
| analgesic/ tetanus serum (which one/ time) |  |
| position of the goat kid |  |
| after-bleeding? |  |
| environmental temperature |  |

**Recovery**

| body temperature (time) |  |
| --- | --- |
| first movement (time) |  |
| sternal (time) |  |
| attempt to stand (time) |  |
| stable standing (time) |  |
| vocalization |  |
| head rubbing (or other signs of pain at disbudded areas) |  |
| surveillance? |  |

**VAS:**

| Observer: |  |
| --- | --- |
| Disbudder: |  |

**other comments:**

|  |  |
| --- | --- |
