# Supplementary material for: Evaluation of anaesthesia and analgesia quality during disbudding of goat kids by certified Swiss farmers
Source: BMC Vet Res. 2018 Jul 9;14:220. doi: 10.1186/s12917-018-1544-7 (PMC6038348; doi:10.1186/s12917-018-1544-7)
Supplement: Supplementary file 2 — Data collection recorded with a standardized protocol (farm protocol). Includes the farm protocol (DOCX 38 kb). [file 12917_2018_1544_MOESM2_ESM.docx]

***Additional file 2***

Data collection recorded with a standardized protocol (farm protocol).

General farm data:

| name |  |
| --- | --- |
| breeds |  |
| farm size |  |
| number of goat/group  group size |  |
| age distribution in groups |  |
| veterinarian |  |

Farmer (dehorning person)

| name |  |
| --- | --- |
| certificate of competence since |  |
| right-/left hander |  |

**Disbudding**

| number of assisting persons |  |
| --- | --- |
| Ø number of goat kids born per year |  |
| Ø number of kids disbudded per year |  |
| number of disbudded goat kids today |  |
| Ø age at disbudding |  |
| disbudding reasons |  |
| assessment of:   - workload - stress for the goat kids - past complications (which ones) - costs |  |

Environment

| anaesthesia induction/recovery area   - infrastructure (bedding, heat lamp, temperature) - surveillance? same area for induction/recovery? awake animals in the same area? |  |
| --- | --- |
| workplace (padding, lightning, power source) |  |
