# Supplementary material for: Evaluation of anaesthesia and analgesia quality during disbudding of goat kids by certified Swiss farmers
Source: BMC Vet Res. 2018 Jul 9;14:220. doi: 10.1186/s12917-018-1544-7 (PMC6038348; doi:10.1186/s12917-018-1544-7)
Supplement: Supplementary file 3 — Description of the behavioural states and events observed during anaesthesia induction and recovery of goat kids before and after disbudding. Describes the following behaviours: Staggering, recumbency, loss of posture, first movement, attempt to stand, steady standing (DOCX 36 kb). [file 12917_2018_1544_MOESM3_ESM.docx]

***Additional file 3***

Description of the behavioural states and events observed during anaesthesia induction and recovery of goat kids before and after disbudding.

| **Behaviour** | **Description** |
| --- | --- |
| Staggering | Goat kid is walking uncoordinated, not able to balance its weight equally over all four limbs |
| Recumbency | Sternal: goat kid is lying in sternal position, still having control of posture  Lateral: goat kid is lying in lateral position, having little control of posture |
| Loss of posture | Goat kid is in a recumbent position with total loss of control of posture |
| First movement | First movement of goat kid after disbudding (transport to recovery area not included) |
| Attempt to stand | First attempt to get up in a standing position (might or might not be successful) |
| Steady standing | Goat kid is able to stand in an upright position with all four legs equally balanced on the floor for a minimum of 60 s without support and/or able to walk several steps without staggering or falling down |
