# Supplementary material for: Evaluation of anaesthesia and analgesia quality during disbudding of goat kids by certified Swiss farmers
Source: BMC Vet Res. 2018 Jul 9;14:220. doi: 10.1186/s12917-018-1544-7 (PMC6038348; doi:10.1186/s12917-018-1544-7)
Supplement: Supplementary file 4 — Description of behavioural events observed during disbudding of anaesthetised goat kids. Describes the following events: Head lifting, limb movement, focused eye movement, spontaneous blinking, ear movement, tail movement, nose movement, mouth movement, vocalisation (DOCX 37 kb). [file 12917_2018_1544_MOESM4_ESM.docx]

***Additional file 4***

Description of behavioural events observed during disbudding of anaesthetised goat kids.

| **Event (Behaviour)** | **Description** |
| --- | --- |
| Head lifting | Head is lifted from the atonic lying position to a more upright position; the head no longer entirely touches the floor |
| Limb movement | Paddling: slowly regular movement of one or more limbs (may be repeated)  Kicking: rapid, forceful thrust with one or several limbs (singular)  Pulling-up limb: goat kid is moving one or more limb towards its body |
| Focused eye movement | Accommodation of the eye (sign of consciousness) |
| Spontaneous blinking | Blinking in absence of palpebral or ocular stimulation |
| Ear movement | One or both ears move spontaneously |
| Tail movement | Tail moves in any direction, no longer lays atonic on the floor |
| Nose movement | Any move of the nose (for example sniffing) |
| Mouth movement | Lips move without vocalisation (for example teeth grinding, chewing) |
| Vocalisation | Sounds made by the vibration of the vocal folds   - Loud Vocalisation: notable vocalisation, except groaning - Weak vocalisation: groan   - Association with painful stimulus (during disbudding)   - No association with painful stimulus (before or after disbudding) |
