# Supplementary material for: Evaluation of anaesthesia and analgesia quality during disbudding of goat kids by certified Swiss farmers
Source: BMC Vet Res. 2018 Jul 9;14:220. doi: 10.1186/s12917-018-1544-7 (PMC6038348; doi:10.1186/s12917-018-1544-7)
Supplement: Supplementary file 5 — General reaction score of goat kids during disbudding based on vocalisation, limb movement and head lifting. A score between 0 and 4, zero means no movement and no vocalisation, four means strong movements and vocalisation (DOCX 36 kb). [file 12917_2018_1544_MOESM5_ESM.docx]

***Additional file 5***

General reaction score of goat kids during disbudding based on vocalisation, limb movement and head lifting.

| **Score** | **Description** |
| --- | --- |
| 0 | No movement and no vocalisation |
| 1 | 1-2 movements (1-2x paddling/kicking or pulling up 1 limb or 1x vocalisation) |
| 2 | Several movements, weak vocalisation (3-6x paddling/kicking or pulling-up one limb and/or 1-2x paddling/kicking or pulling-up several limbs and/or 2-6x vocalisation) |
| 3 | Strong movements and vocalisation (more than 6x paddling/kicking or pulling-up one limb and/or more than 2x paddling/kicking or pulling-up several limbs and/or more than 6x vocalisation and/or occurrence of head lifting) |
