# Supplementary material for: Evaluation of anaesthesia and analgesia quality during disbudding of goat kids by certified Swiss farmers
Source: BMC Vet Res. 2018 Jul 9;14:220. doi: 10.1186/s12917-018-1544-7 (PMC6038348; doi:10.1186/s12917-018-1544-7)
Supplement: Supplementary file 6 — Risk factors for the three outcomes grade, first movement and steady standing, divided in farm-level risk factors and goat kid-level risk factors. Description and categorization of the different risk factors on farm and goat kid level (DOCX 38 kb). [file 12917_2018_1544_MOESM6_ESM.docx]

***Additional file 6***

Risk factors for the three outcomes grade, first movement and steady standing, divided in farm-level risk factors and goat kid-level risk factors.

| **Risk factor** | **Description/ categorization** |
| --- | --- |
| ***Farm level*** |  |
| Herd size | < 30 dairy goats, 30 -100 dairy goats, >100 dairy goats |
| Average disbudded goat kids per year | < 10, 10-20, > 20 goat kids |
| Heat lamp during induction | yes/no |
| Heat lamp during recovery | yes/no |
| Presence of awake goats not undergoing disbudding procedure during induction or recovery | yes/no |
| Disbudding technique | Dehorner slid around *versus* upright on skull  Pen knife/shear used before thermal disbudding: yes/no  Horn bud removed after disbudding: yes/no  Mean burning time per side: < 20 s, 20-60 s, > 60s  Number of times applying dehorner (per side): < 2, 2−4, > 4 times  Restraint of the goat kid during disbudding: yes/no |
| Environmental temperature | During induction: < 15°C, > 15°C  During recovery: < 15°C, > 15°C |
| ***Goat kid level*** |  |
| Breed | Chamois-Coloured Goat, Saanen, Toggenburg, [Grisons Striped](https://en.wikipedia.org/wiki/Grisons_Striped) |
| Age | < 14 days, > 14 days |
| Weight | < 6 kg, > 6kg |
| Health situation | good/not good |
| Sex | female/male |
| Off-feed time | < 1 h, 1-3 h, 3-6 h, > 6 h |
| Anaesthetic drug | 1. Xylazine and ketamine mix as recommended in the course 2. Xylazine and ketamine mix in dosage other than recommended 3. Xylazine alone 4. Xylazine and local lidocaine 5. Acepromazine and ketamine |
| Analgesic (meloxicam/ tolefenamic acid) administered | yes/no |
| Injection site | forelimb, hindlimb, neck |
| Aspiration before injection | yes/no |
| Time from injection to beginning disbudding | < 5 min, 5-10 min, 11-20 min, > 20min |
| Intervention of farmer during induction | yes/ no |
| Intervention of farmer during recovery | yes/ no |
| Additional dosage of drugs administered | yes/ no |
